# Delivering ‘tiny targets’ in a remote region of southern Chad: a cost analysis of tsetse control in the Mandoul sleeping sickness focus

**DOI:** 10.1186/s13071-020-04286-w

**Published:** 2020-08-14

**Authors:** Jean-Baptiste Rayaisse, Fabrice Courtin, Mahamat Hisséne Mahamat, Mahamat Chérif, Wilfrid Yoni, Nadmba M. O. Gadjibet, Mallaye Peka, Philippe Solano, Steve J. Torr, Alexandra P. M. Shaw

**Affiliations:** 1 Centre International de Recherche-Développement sur lʼElevage en zone Subhumide (CIRDES), Bobo-Dioulasso, Burkina Faso; 2 Institut Pierre Richet (IPR), Bouaké, Côte d’Ivoire; 3 Institut de Recherche en Elevage pour le Développement (IRED), NʼDjaména, Chad; 4 Programme National de Lutte contre la Trypanosomiase Humaine Africaine (PNLTHA), N’Djaména, Chad; 5 Institut de Recherche pour le Développement (IRD), Unité mixte de recherche, (UMR) 177 Intertryp IRD-Centre de coopération internationale en recherche agronomique pour le développement (CIRAD), Université Montpellier, Montpellier, France; 6 Liverpool School of Tropical Medicine (LSTM), Liverpool, Merseyside UK; 7 Infection Medicine, Deanery of Biomedical Sciences, College of Medicine and Veterinary Medicine, The University of Edinburgh, Edinburgh, UK; 8 AP Consultants, Walworth Enterprise Centre, Andover, UK

**Keywords:** Cost, Tsetse control, Tiny targets, Human African trypanosomiasis, Chad, Mandoul

## Abstract

**Background:**

Since 2012, the World Health Organisation and the countries affected by the Gambian form of human African trypanosomiasis (HAT) have been committed to eliminating the disease, primarily through active case-finding and treatment. To interrupt transmission of *Trypanosoma brucei gambiense* and move more rapidly towards elimination, it was decided to add vector control using ‘tiny targets’. Chad’s Mandoul HAT focus extends over 840 km^2^, with a human population of 39,000 as well as 14,000 cattle and 3000 pigs. Some 2700 tiny targets were deployed annually from 2014 onwards.

**Methods:**

A protocol was developed for the routine collection of tsetse control costs during all field missions. This was implemented throughout 2015 and 2016, and combined with the recorded costs of the preliminary survey and sensitisation activities. The objective was to calculate the full costs at local prices in Chad. Costs were adjusted to remove research components and to ensure that items outside the project budget lines were included, such as administrative overheads and a share of staff salaries.

**Results:**

Targets were deployed at about 60 per linear km of riverine tsetse habitat. The average annual cost of the operation was USD 56,113, working out at USD 66.8 per km^2^ protected and USD 1.4 per person protected. Of this, 12.8% was an annual share of the initial tsetse survey, 40.6% for regular tsetse monitoring undertaken three times a year, 36.8% for target deployment and checking and 9.8% for sensitisation of local populations. Targets accounted for 8.3% of the cost, and the cost of delivering a target was USD 19.0 per target deployed.

**Conclusions:**

This study has confirmed that tiny targets provide a consistently low cost option for controlling tsetse in gambiense HAT foci. Although the study area is remote with a tsetse habitat characterised by wide river marshes, the costs were similar to those of tiny target work in Uganda, with some differences, in particular a higher cost per target delivered. As was the case in Uganda, the cost was between a quarter and a third that of historical target operations using full size targets or traps. 
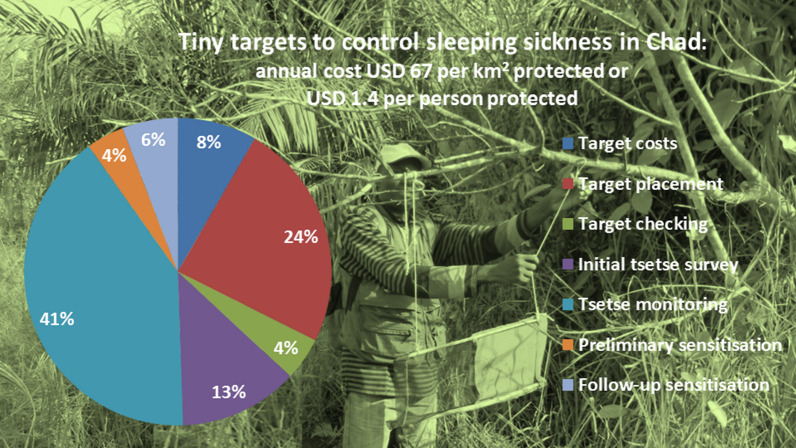

## Background

Since about 2005, a growing body of evidence on the costs of tsetse control based on field operations has been gathered and published. This reflects the renewed focus on both human and animal African trypanosomiasis (HAT and AAT). For HAT, this followed the recognition that the continent was once again facing an epidemic of the disease [1]. The World Health Organization (WHO) and the affected countries have made a commitment to achieving elimination as a public health problem by 2020 of the gambiense form of the disease, which occurs in West and Central Africa and is caused by *Trypanosoma brucei gambiense* [2, 3]. They further aim to achieve zero incidence by 2030 [4] and substantial progress towards achieving this has been made [5]. Recent papers include some comprehensive cost analyses [6–8] as well as articles focusing on key cost components [9–12]. These cost studies have been included in an analysis of the historical costs of using fixed and mobile baits and aerial spraying, updated to current prices [13]. However, important gaps remain. Few analyses of the costs of tsetse control examine the full costs of the interventions, often concentrating on core costs such as traps, targets, flying time. Most relate to schemes targeting AAT. However, a systematic review of those targeting HAT has been undertaken [14] as well a brief analysis of the cost of traps and targets used for HAT control [8]. For Chad, cost analyses include a description of groundspraying in the late 1970s [15] and of the use insecticide footbaths for cattle to control AAT and eventually impact on the transmission of HAT [12]. The only previously recorded use of vector control in a Chadian HAT focus is a mention of a pilot project using 77 targets undertaken in 1987/88 in the Tapol focus, located west of Moundou about half-way to the Cameroonian border [16].

The Mandoul focus’s history mirrors the wider picture of HAT in Africa. It was first described in 1912 in the Moissala area [17]. Gaston Muraz identified an outbreak in the Mandoul region, near Bodo in 1928 [18, 19]. Its history and resurgence are described [20]. During the 1960s the incidence of HAT was reduced to very low levels, with only four cases reported in the whole of Chad in 1975, so that HAT was thought to have virtually disappeared from the area. However, in 1993 the focus had become active again with unpublished results of a survey by F. Boyer showing a prevalence of 4.6% among the population tested, rising to the very high levels of 11.8% and 17.6% in two villages. Active surveillance was implemented and by 1999 the prevalence among the population thus examined for HAT had fallen to 0.44%. No active surveillance was undertaken in 2000 and 2001; however, returning to the area in 2002/2003, 2.5% of people screened were found to have HAT and 715 cases were reported. Thereafter intensive active surveillance continued and the number of cases fell to 186 in 2013. However, this level was still well above the WHO’s threshold for the elimination of HAT as a public health problem, which is set at 1 case per 10,000 people [21]. For the population of the Mandoul focus, this represented 3.9 cases per 10,000 people. In 2010 the government of Chad had resolved to tackle the vector, and designated the Mandoul focus as a priority area for vector control, as it accounted for the overwhelming majority of HAT cases in Chad. This initiated the project being analysed in this paper, which began at the end of 2013 [21]. The number of cases recorded fell to 90 in 2014 and 47 in 2015 [21].

The cost of using tiny targets to control tsetse in a HAT focus was analysed for Uganda [8] and this was incorporated in an economic analysis of the prospects for the elimination of HAT as a public health problem undertaken for the WHO [22]. The tiny target technology is also being deployed in HAT foci in Chad, Côte d’Ivoire, the Democratic Republic of Congo and Guinea. In order to understand how the costs of these operations vary in different organisational, demographic and ecological contexts, a cost analysis was undertaken in Chad, refining the data collection protocol developed for the Uganda study. A previous study described the impact of tiny targets on tsetse and the incidence of sleeping sickness in the Mandoul focus [21].

With the extension of the tiny target technology to control tsetse in other HAT foci in Africa, it is essential to have an idea of how consistent the costs of implementing it are in different settings. The objective of this study was to refine the cost data collection approach used in Uganda and to use it to track the costs of using tiny targets in a contrasting situation. Chad’s Mandoul focus differs from Uganda in its tsetse habitat (wide swampy wetlands as compared to narrow fringing vegetation), the project’s organisational structure (a local research and development organisation as against an externally led research project) and its location (a remote area compared to one with relatively easy access to a major town, Arua, in Uganda).

## Methods

### Study area

**Fig. 1:**
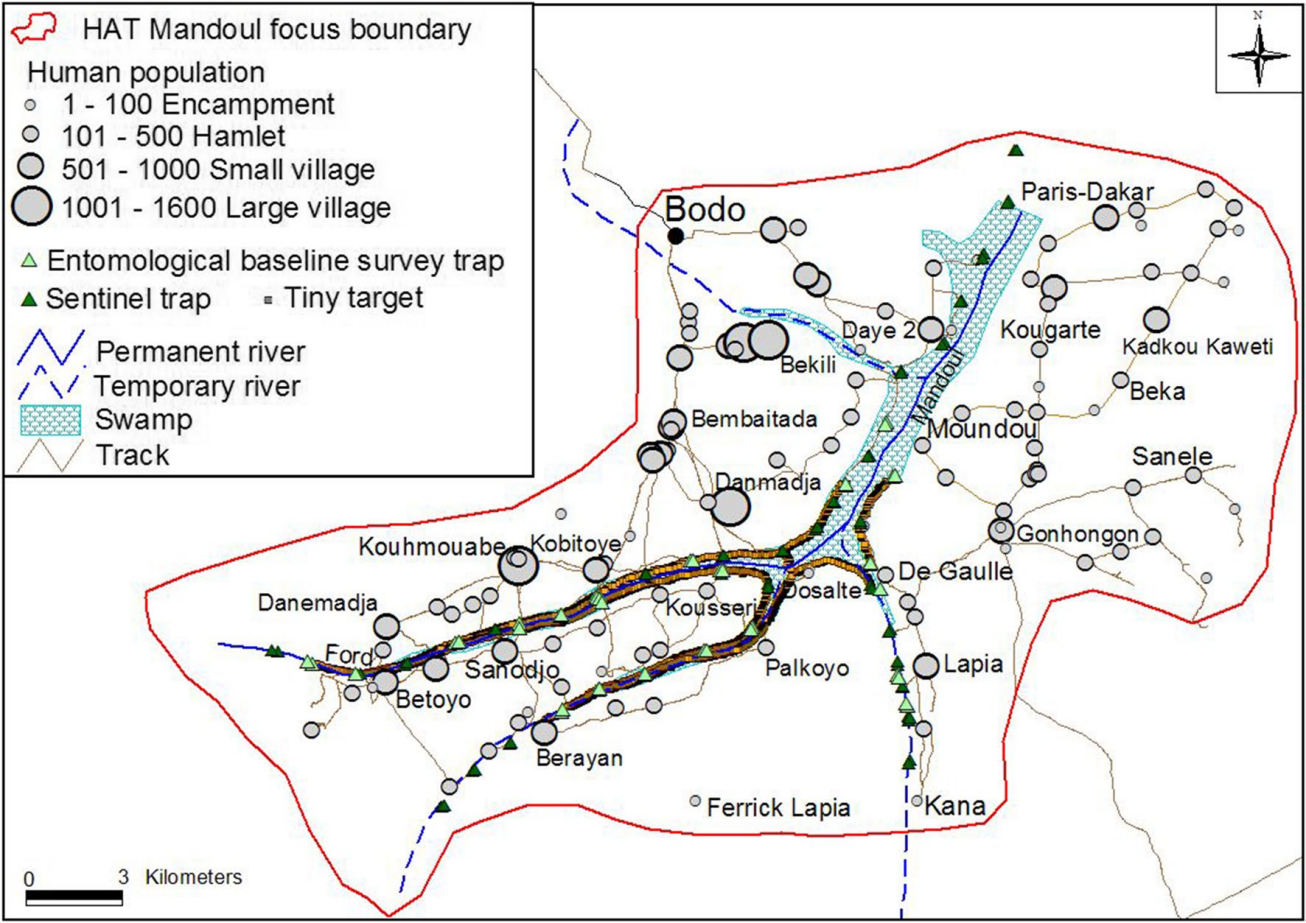
The Mandoul HAT focus

**Fig. 2:**
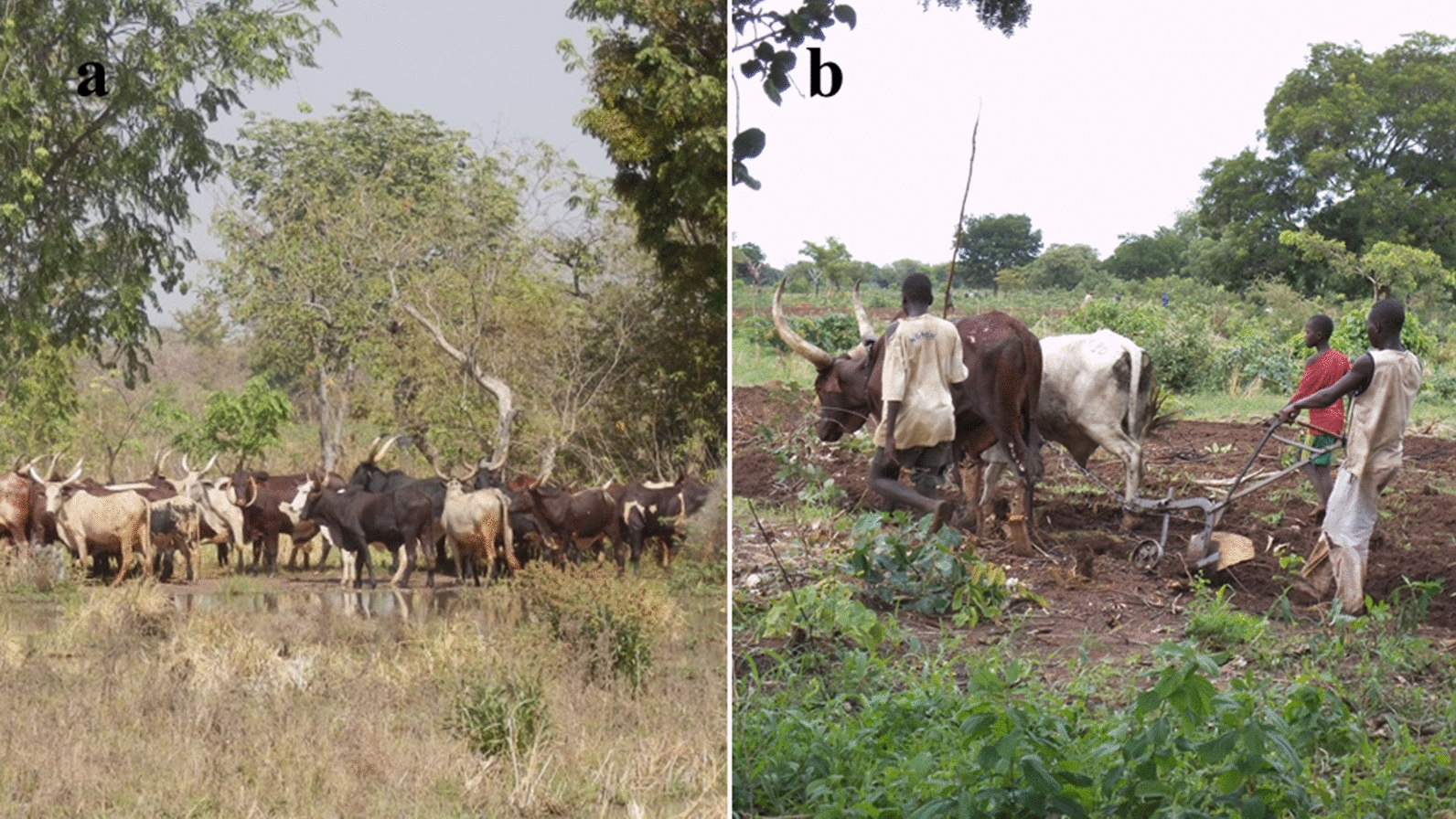
Cattle in the Mandoul HAT focus. **a** Mbororo transhumant cattle grazing in marshland. **b** Sedentary cattle used for animal traction

The Mandoul HAT focus (Fig. 1) is located in southern Chad, southeast of the town of Moundou and within 100 km north of the border with the Central African Republic. The boundaries of the focus were drawn by taking into account several factors, notably the presence of water courses and of favourable biotopes for tsetse flies using satellite maps, the occurrence of HAT cases in the recent past, the areas from which people and livestock move in and out of the tsetse habitat and the presence of tsetse. The latter was based on historical data as well as the 2013 tsetse survey, yielding an area of 840 km^2^ ‘protected’ by the intervention along the banks of the Mandoul swamp. A population census was conducted as part of the tsetse control project. This mapped the human settlements of different sizes (Fig. 1) and the number of inhabitants as well as counting the livestock in the area. From this it was calculated that the focus contains 38,674 people or 46 per km^2^. Of this population, 52% live in 27 villages of over 500 inhabitants and the remainder in 72 encampments and hamlets [21]. Farming and livestock keeping are the predominant activities (growing sorghum, millet, as well as cotton and market gardening), with 13,863 sedentary cattle kept in the area year-round and 2954 pigs enumerated during the census. The left (northwestern) bank of the Mandoul swamp which includes the town of Bodo, contained 68% of the people, 60% of the cattle and 72% of the pigs. Cattle are used for ploughing and, especially on market days, for pulling carts. Just over half the cattle on the right (southwestern) bank are in the five ferricks, owned by Mbororo, Fulani and Arab pastoralists (Fig. 2). During the dry season, parts of the northern Mandoul river bed dry up and are grazed by livestock owned by transhumant Mbororo pastoralists [23].

Historical tsetse surveys found both *Glossina fuscipes fuscipes* and *Glossina morsitans submorsitans* to be present in the Mandoul area [24, 25] but no tsetse control activities were undertaken. Another recent study undertaken in the Moundou and Sahr areas, respectively located some 150 km to the east and west of the Mandoul focus, found *G. tachinoides* as well as *G. f. fuscipes* and in Sahr, *G. m. submorsitans* was also present [12]. Surveys undertaken in Mandoul by the project in November 2012 found no *G. m. submorsitans* leading to the conclusion that increasing human population, with the attendant habitat and large game population reduction had led to its disappearance [21], as discussed elsewhere [26]. The extensive ‘T0’ baseline tsetse survey conducted in November 2013 before the current target-based tsetse control programme began, also found no *G. m. submorsitans*, confirming that *G. f. fuscipes* is the only species present [21].

### Data collection

Following discussions and pre-project planning, field work began at the end of October 2013, with the initial tsetse survey of the Mandoul focus. The first target deployment took place in January 2014, and has continued annually since then. The cost study was initiated at the end of 2014. It was based on the methodology developed and tested in the cost analysis of the tiny target project undertaken in Uganda [8]. A detailed new cost collection protocol was written up and a simple Microsoft Excel® spreadsheet created for recording the costs incurred during each field mission under the following headings: (i) activity report including dates, team leader, narrative, productivity indicator (e.g. number of targets deployed) and work category (tsetse survey/monitoring, sensitisation, target deployment or target maintenance); (ii) personnel (people involved, work dates and days, travel allowances paid); (iii) vehicle and transport costs (fuel, vehicle and canoe hire); (iv) consumables and equipment for field work; (v) administrative overheads (office equipment and salaries of staff involved in the project).

The additional files include copies of the data collection protocol in both English and French (Additional file 1: Text S1 and Additional file 2: Text S2), the blank spreadsheet used also in both languages (Additional file 3 and Additional file 4) as well as the completed basic spreadsheet (Additional file 5).

Data on all project field activities and expenditures was collected for two full calendar years, 2015 and 2016. It was completed using previously recorded information on data on the cost of the initial tsetse survey and preliminary sensitisation work in 2013. In order to cross-check mileages and vehicle use, cost data on the 2014 and 2018 deployments were also investigated. The central collection point for costs was the Institut de Recherche en Élevage pour le Développement (IRED).

### Economic analysis of costs

#### Full cost approach

The approach used in this assessment of the costs of tiny targets is essentially the same as described in [8] for Uganda. The costs calculations aim to represent the full cost to society of implementing tsetse control using tiny targets. Thus, firstly, the costs need to go beyond the cost borne by individual institutions, to include the contributions made by all stakeholders, in the case of this project: IRED, the Chadian Programme National de Lutte contre la Trypanosomiase Humaine Africaine (PNLTHA) and Centre International de Recherche-Développement sur lʼElevage en zone Subhumide (CIRDES) as well as contributions from local communities in the tsetse control zone. The costs also go beyond quantifying the incremental budget required to undertake the work to looking at all resources used, using the ‘full cost’ approach [13, 27]. In practical terms this means estimating the shares of salaries, administrative overheads, office costs and depreciation on equipment used for the project, whether bought for it or already present in the organisations involved.

#### Research and control components

Secondly, the objective is to work out the costs of a control, rather than a research operation. The tiny target projects undertaken to control HAT in Chad, Côte d’Ivoire, Democratic Republic of Congo, Guinea and Uganda [21, 28, 29] are pioneering interventions which all have a strong research component. The costs as calculated here and for Uganda [8] aim to produce values which can be extrapolated to other situations as part of the programme for the elimination of gambiense HAT. As noted above, the costing work done in Uganda has already helped to inform such an analysis [22]. Accordingly, the expenditures recorded were adjusted so as to remove the purely research components of the project such as laboratory materials or the costs of PCR and other tests performed on return from field work.

In addition to IRED, which managed the project, the other Chadian organisation involved was the PNLTHA which is responsible for surveillance, testing and treating human patients. Including both the Ministries of Health and Animal Resources was a key principle for the project. The two tsetse control specialists and the geographer were based in Burkina Faso, at CIRDES and the Institut de Recherche pour le Développement (IRD). Specialists from the Liverpool School of Tropical Medicine (LSTM) provided oversight and visited the project site to conduct additional research activities.

In order to provide costs which are applicable to a locally run non-research-based operation, in the cost calculations staff salaries were capped at rates applicable for IRED medium grade research staff, CFA 500,000 per month (USD 843) and per diems for field travel involving nights away from base at CFA 30,000 per day (USD 50). However, for the administrative staff based at IRED headquarters in Chad, actual salaries were used. Similarly, for researchers travelling to Chad from other countries, international travel time and costs were excluded. A research evaluation undertaken by researchers from LSTM, studying the uptake of messages on tsetse control from the sensitisation activities was also left out of the cost calculations.

#### Prices

During the two-year period analysed, there was little inflation, with expenditures on key items remaining very similar. Thus actual costs were used, converted to United States dollars (USD) from central African BEAC (Banque des États de lʼAfrique Centrale) CFA Francs (XAF) at the average rate of USD 1 = F CFA 593, the rate applying for the calendar year 2016. Both the west and central African CFA francs are pegged to the Euro at a rate of EUR 1 = CFA 656. In line with the convention that has prevailed in all tsetse and trypanosomiasis cost calculations and publications, the prices used are local, in-country, market prices, also called nominal prices. There was no adjustment for purchasing power parity to convert costs to international dollars. The costs calculated thus provide a transparent estimate which readers from other areas can adjust for their price levels. In the discussion, where USD prices from other studies were cited, these were converted to 2016 price levels using the mid-year US inflation rate which can be obtained at http://inflationdata.com/inflation/Inflation_Rate/HistoricalInflation.aspx.

#### Capital items

As the calculations cover a two-year period, with the objective of calculating an annual cost, the cost of capital items was reflected by an estimate of depreciation, based on how long their useful life was likely to be and what share of this was being used up in a particular activity. Targets are all replaced annually. Traps were estimated to be useable for four years, based on the monitoring activities with three field trips a year and traps being deployed at 2 or 3 sites. Vehicles were generally hired, so no depreciation was calculated. The hire included a driver, and the prices ranged between CFA 50,000 and 70,000 per day (USD 84–118), averaging USD 98 per day. For consistency, this daily rate was applied on the few occasions when vehicles belonging IRED were used. Four global positioning system (GPS) sets belonging to the research institutes were used during target deployment, two for tsetse monitoring and target checking and none for sensitisation (public awareness) work. Their useful life was estimated at five years, with only about a third of their annual use being on the Chad project.

For the administrative and office overheads, all the furniture, computer and electrical items present at the IRED offices in 2015 were valued, coming to a total of USD 21,920. A useful life of four years was allocated for all items. Project use was estimated at 8% of annual use, with the exception of the electric generator which was only used for the project, and the computers/laptops. Depreciation was calculated on a computer for each person working on the project whether in the field or in the office, in proportion to the total time they spent on the tiny targets project. These costs came to USD 766 per annum and were added to the other administrative costs and allocated to the project activities in proportion to the time required for field work.

Lastly, the initial tsetse survey (called T0 in the project) and preliminary sensitisation work undertaken in 2013 are also treated as ‘investments’, to be depreciated over the life of the project. Although target deployment is still ongoing, it was decided to spread their cost over five years of control work (2014–2018), thus allocating 20% of the total cost of the initial T0 tsetse survey and the initial sensitisation to the costs actually incurred in each year.

#### Staff

Field staff costs consisted of travel allowances and salaries. The researchers and technicians received their salary and a per diem payment for time away on field trips of 30,000 FCA (USD 50) to cover all costs. Based on weekend and holiday entitlements, a working year of 240 days was assumed. In order to calculate the value of salaries it was estimated that in addition to time in the field, the head of the team required three days to prepare for each mission and the other members of the team, two days, reducing to two and 1.5 days, respectively, for the shorter sensitisation missions. Thus, for each team member the total number of days worked was calculated, then divided by 20 to obtain the number of salary months, which were then ascribed to each activity.

#### Administrative support

A project of this nature also needs administrative staff support. The 2015–2016 costs were based on a month’s time per year for the accountant and a member of staff helping with the cost data collection, plus inputs of a few days per year from the director general, the director’s secretary and the department head along with a lump sum to cover cleaning, guards and other support staff. This came to a total of USD 2242 per annum. Office running costs were recorded for rent, stationery, telephone, internet, electricity and water and an appropriate share allocated to the tsetse control work, coming to USD 1987 per annum. These figures were added to the depreciation on office furniture and equipment and then shared out between the activities in proportion to the staff time allocated to each, under the heading “administrative support”.

Thus, by including administrative overheads, a share of salaries, while removing purely research costs, it is hoped that the costs calculated represent a robust estimate of the full economic cost of mounting this control operation. The results are set out below, starting with details of staff and timing and then going chronologically through the different activities.

## Results

### Timing of activities

**Fig. 3:**
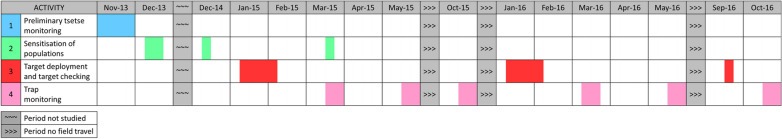
Schedule of work for which costs were collected and analysed

The activities included in the costing exercise and their timing over the period from November 2013 to October 2016 are illustrated in Fig. 3. The duration of the field work and a brief description are provided in Table 1. Although the field time varied slightly from mission to mission, as did the number of people involved, the timings were similar from year to year. Monitoring was undertaken three times a year, for a two week period. Deployment work started in the second week of January and finished early February, with an additional maintenance check on the targets being undertaken in September 2016. The sensitisation work was undertaken at the start of the period studied, then not repeated until 2017, which was after the cost monitoring had ended.

**Table 1:** Timing and duration of field activities

Activity	Timing	Trip duration (days)	Description
Preliminary tsetse survey T0	October–November 2013	30	Traps deployed at 108 sites
Initial sensitisation	December 2013	15	Radio spot produced and broadcast. Creation of CSSEs^a^
Period before cost monitoring started	January–November 2014	–	First deployment January 2014, trap monitoring T1, T2 and T3 in March, May and October 2014
Re-sensitisation	December 2014	9	Sensitisation campaign and revitalization of CSSEs
Target redeployment	January-February 2015	17	2600 targets replaced and 108 extra deployed, making a total of 2708
Trap monitoring T4	March–April 2015	15	Traps deployed at 44 sites
Sensitisation	March 2015	8	Sensitisation targeting transhumant pastoralist populations
Trap monitoring T5	May 2015	14	Traps deployed at 44 sites
Trap monitoring T6	October 2015	13	Traps deployed at 44 sites
Target redeployment	January–February 2016	25	2708 targets replaced
Trap monitoring T7	March 2016	11	Traps deployed at 44 sites
Trap monitoring T8	May 2016	17	Traps deployed at 44 sites
Target check	September 2016	7	Placement and condition of deployed targets checked
Trap monitoring T9	October 2016	13	Traps deployed at 44 sites

^a^ “Comités de suivi et de sauvegarde des écrans”, committees for monitoring and safeguarding of targets

### Human resources

The field work was undertaken by researchers and technicians from the five institutes, IRED, PNLTHA, CIRDES, IRD and LSTM. The total time input is summarised in Table 2, with a total number of 327 person days for the preparatory work in 2013 and 236 and 278 days respectively in 2015 and 2016.

**Table 2:** Field travel days for staff from the research institutes

Activity	2013^a^		2015^b^		2016	
	No. of staff	Person days	No. of staff	Person days	No. of staff	Person days
Tsetse surveys and monitoring^**c**^	9	282	3	128	3–4	151
Sensitisation	5	75	2	34	–	–
Deployment	–	–	4	74	4	94
Maintenance	–	–	–	–	5	33
Total	–	357	–	236	–	278

^a^Preparatory work before target deployment undertaken

^b^Sensitisation to prepare for the January 2015 target deployment was undertaken in December2014

^c^There were three monitoring field trips each year in 2015 and 2016

Once in the intervention zone, local people were recruited for a range of activities. Key to the success of the project were the “Comités de Suivi et de Sauvegarde des Ecrans” (CSSEs) which were created during the initial sensitisation campaign in December 2013. These were local teams of four individuals per village, and were set up to help with the target deployment and their surveillance. They received incentive payments and were re-involved as the project progressed, for example during the second sensitisation in December 2014 and the deployment in January 2016, when 195 CSSE members participated. During most of the field trips, up to 4 CSSE members, also acting as guides, participated in the field work and paid CFA 2000–2500. Likewise, local supervisors, who had been involved with the medical team for a long time, accompanied the entomological team during all types of activities and were paid CFA 5000 (USD 8) per day. Occasionally a guard was also hired and local four field assistants joined the team for the 30 days of the initial tsetse survey. The average annual expenditure on local labour came to USD 1408, or 2.5% of costs.

**Fig. 4:**
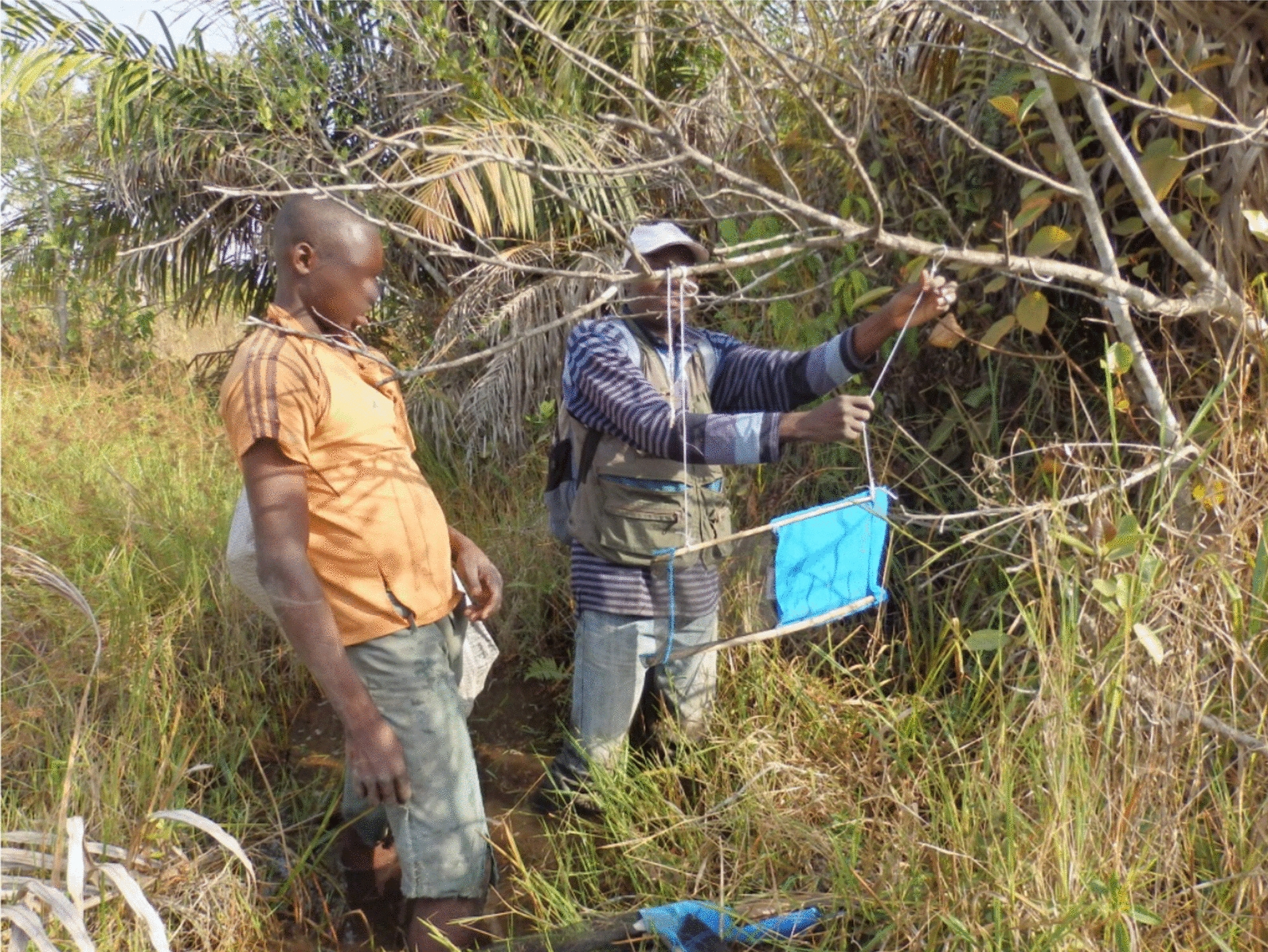
Target deployment

Staff training was undertaken in the field (Fig. 4). Researchers and technicians from IRED already had some familiarity with the techniques being used and local supervisors and CSSE were introduced to the tsetse control methods during the sensitisation activities, and received further training in the field.

### Tsetse survey and monitoring

**Fig. 5:**
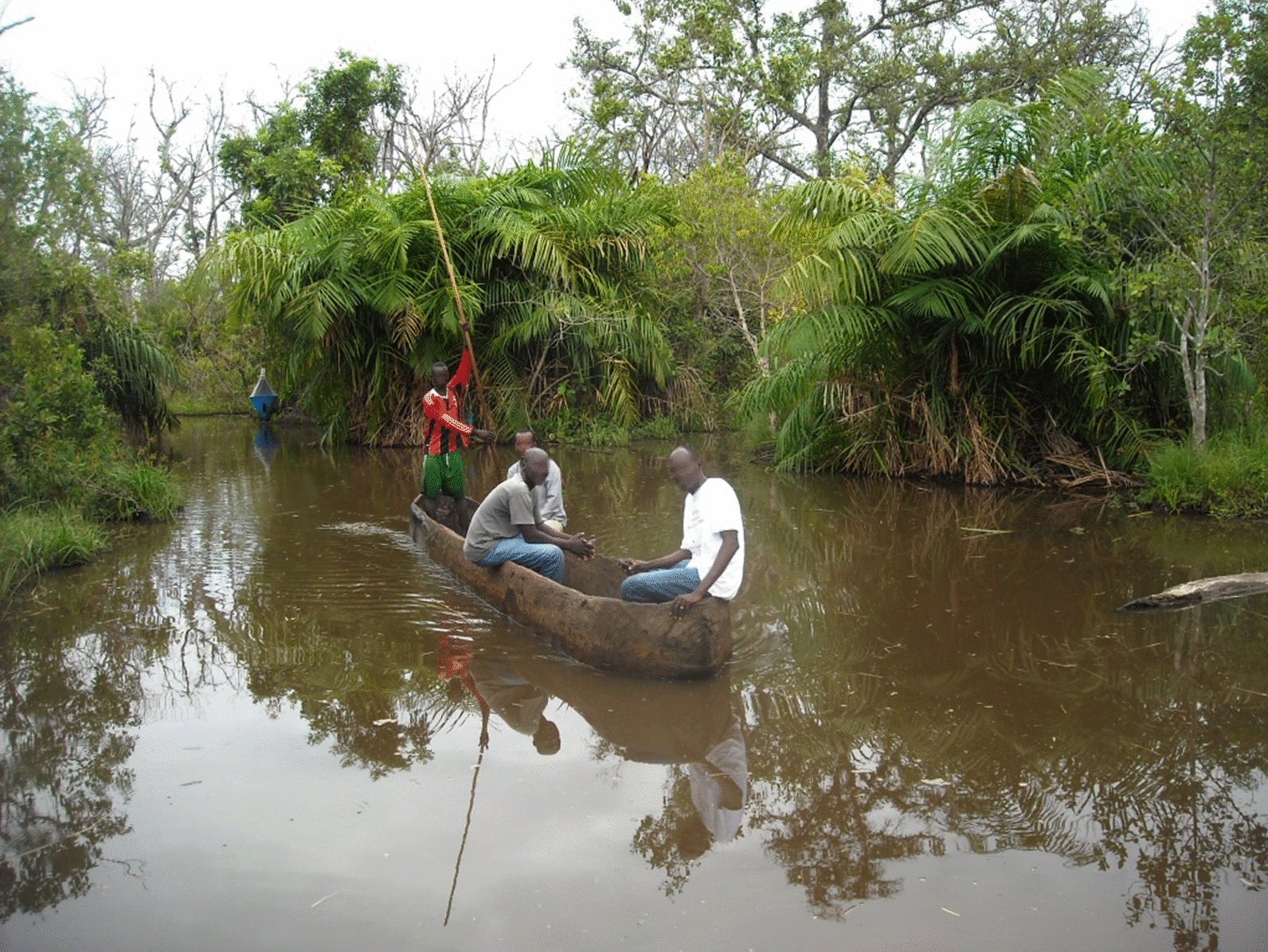
Accessing target deployment and trap monitoring sites by canoe (note the biconical trap just behind the canoe)

Once the basic organisational framework was in place, the project’s first activity was to conduct an extensive baseline tsetse survey. This was undertaken in November 2013, using biconical traps (see centre background of Fig. 5). These were deployed for 48 hours at 108 sites [21] which included the sites later used for sentinel traps (Fig. 1). The costs are given in Table 3, and came to USD 35,977, which as explained above, provided the baseline information for five years of deployment, so that in the final cost calculations, 20% of this cost or USD 7195 is allocated to each year’s work, coming to USD 8.6 per km^2^. These T0 costs excluded the field costs for fly dissection and analysis (USD 826 of laboratory materials and USD 165 for tables and chairs) as these were purely for research.

**Table 3:** Cost of initial tsetse survey and subsequent monitoring using traps

Item (percentage of average monitoring round’s costs)	2013 Initial tsetse survey (USD)	2015 Monitoring (USD)	2016 Monitoring (USD)	Average cost per monitoring round (USD)
Specialised equipment (0.6%)				
Traps (depreciation)^a^	114	126	126	42
Trap cages and sleeves	14	24	14	6
Transport (24.2%)				
Vehicle hire^b^	2403	3946	3794	1293
Fuel	2040	2000	1235	539
Vehicle maintenance charge and repairs^b^	582	–	–	–
Canoe hire	–	9	24	6
Motorcycle hire	540	–	17	–
Staff (58.1%)				
Share of staff salaries	11,047	5464	6223	1948
Travel allowances	13,153	6476	8331	2468
Local labour (2.1%)				
Supervisors, guards and field assistants	354	498	438	156
Village chiefs	337	–	–	–
Administrative support (11.8%)	3996	2697	2697	899
Consumables and equipment (3.2%)				
GPS sets (depreciation)	82	122	122	41
GPS sets batteries	71	101	57	26
Fuel for generator^c^	303	191	–	32
Electrical supplies, hammers and stationery	339	–	–	–
Gas bottle refills, grease	30	16	28	7
Sundries (including internet, medical supplies)	571	420	376	133
Total	35,977	22,090	23,482	7595
Cost per km^2^ protected	42.8	26.3	28.0	9.0

^a^30 traps were used for regular monitoring and 45 for the initial tsetse survey

^b^For the initial survey two IRED vehicles were used, in addition to the hired vehicle. Thus, hire charges were estimated as explained under methods and the costs include a repair plus the lump sum running cost charge made for using those vehicles

^c^During 2016 the local area electricity generator was functional, so no generator was needed by the team

During control operations, monitoring the tsetse populations at regular intervals is essential. For this operation, monitoring lasting about two weeks was undertaken three times a year in March, May and October at 44 selected sentinel trap sites (Fig. 1), where traps were again deployed for 48 hours [21]. In addition to the main vehicle carrying staff, canoes were regularly hired to set traps and targets all along the waterways linking the two branches of the Mandoul River (Fig. 5).

The costs are summarised in the last three columns of Table 3. The monitoring costs were very similar in the two years and averaged at USD 27.1 per year per km^2^ protected. The last column shows what the average cost of a single monitoring round was, just under USD 7600 or USD 9.0 per km^2^.

### Sensitisation

Sensitisation of local populations is essential to the success of any tsetse control operation. Its objective is to ensure that populations (including local administrative staff, religious and traditional leaders) know about the disease and how it is transmitted. It shows them the different tools of control and above all, aims to gain their commitment in terms of effective participation to the field activities. In this project this consisted of three activities: (i) producing a radio spot in three languages (Arabic, French and Ngor) and broadcasting this on the local radio stations Voix du Paysan (VDP), Radio Communautaire de Koumra (RCK) and Radio Takadji de Bodo (RTB); (ii) visiting villagers and transhumant pastoralists and distributing leaflets and posters; and (iii) creation of the CSSEs as described above (under human resources).

The radio spot for broadcasting was put together during the preliminary sensitisation work in December 2013. It was broadcast three times daily (morning, noon and evening) for ten days, just before the first deployment started in January 2014. The CSSEs were also set up during this first mission. The cost came to USD 11,117 (Table 4). As with the initial tsetse survey, 20% of this cost was ascribed to each year of deployment, thus adding an average of USD 2.6 per km^2^ to the annual cost of the programme.

**Table 4:** Cost of preliminary sensitisation mission in 2013 and follow up operational missions in 2014 and 2015

Item (percentage of total costs for 2014/2015 work)	2013 (USD)	2014/2015 (USD)
Specialised equipment (3.0%)		
Distribution of leaflets and posters	169	67
Creation of radio broadcast in Arabic, French and Ngor	422	–
Cost of broadcasting on local radio stations	379	127
Transport (34.2%)		
Vehicle hire	1475	1762
Fuel	292	479
Staff (50.1%)		
Share of staff salaries	3238	1560
Travel allowances	3794	1720
Local labour (1.2%)		
Supervisor	278	76
Administrative support (10.7%)	982	688
Consumables and equipment^b^ (0.9%)		
Fuel for generator	71	61
Total	11,117	6551
Cost per km^2^ protected	13.2	7.8

^a^Posters were only used in 2015

^b^GPS were sets not used for sensitisation field work

At the end of 2014, further sensitisation work was undertaken. The CSSEs were revitalised: contacted and made aware of the upcoming second target deployment in January 2015. The radio spot was again broadcast, but only twice daily, for between three and seven days. As the CSSEs were village-based, it was felt that further outreach was required to inform the transhumant pastoralist populations who migrate into the area during the dry season with their stock. Furthermore, it became evident that the hanging targets were sometimes displaced by cattle, which caught them in their horns when going to the marsh to drink. Thus, in March 2015, it was decided to inform pastoralists about the tiny target operation and distribute leaflets to them. The cost of these two operations came to USD 6551working out at USD 7.8 per km^2^ (Table 4).

### Target deployment

Tiny targets were purchased from Vestergaard, at a standard price of USD 1 each for the manufactured insecticide-impregnated cloth and netting component. While the unit cost of the target fabric was fixed, the cost of freight and insurance varied greatly from order to order [8]. All target orders for the Chad project were airfreighted to N’Djaména from Vietnam. For the 2014 and 2015 shipments of 3000 targets each, which were used for the 2015 and 2016 deployments, the insurance and air freight added 35% and 31%, respectively, to the total cost. The local teams cut sticks for mounting the targets: 4 per standing target and 2 per suspended target, as the majority of targets were hung from branches of vegetation bordering the swamp rather than planted in the ground (Fig. 4). The cost varied from year to year, depending on the contract agreed. Additional costs for assembling the targets were for string, wire and glue. Thus the total cost for each assembled target averaged over the two operations was USD 1.56, of which USD 1.00 was the price of the target, USD 0.33 the insurance and freight and USD 0.23 assembly and materials. The targets accounted for a quarter of the total cost of the deployment activity. Another feature of the deployment work was the use of canoes in greater numbers than for monitoring (Fig. 5).

Targets were deployed in riparian vegetation along the swamp and at crossing points at a density of about 60 targets deployed per linear km [21], as illustrated in Fig. 1. The deployment cost varied from year to year, with the average annual cost coming to USD 18,110 and the cost per km^2^ protected to USD 21.6, of which just over a quarter was for targets (Table 5).

**Table 5:** Cost of deploying targets

Item (percentage of two years’ total costs)	2015 (USD)	2016 (USD)
Specialised equipment (25.8%)		
Targets^a^	3000	3000
Target insurance and freight	1050	930
Target assembly and fixing: cutting up 12,000 sticks and assembling of targets	506	101
String, wire and glue	452	295
Transport (14.4%)		
Vehicle hire	1973	2108
Fuel	422	547
Canoe hire (5 days, from several jetties)	84	84
Staff (43.0%)		
Share of staff salaries	3188	3895
Travel allowances	3744	4755
Local labour (3.8%)		
Supervisor, guard	110	599
CSSEs incentive payments	–	658
Administrative support (9.1%)	1599	1698
Consumables and equipment (3.9%)		
GPS sets (depreciation)	68	68
GPS sets batteries	101	51
Fuel for generator	49	74
Digital camera (depreciation)	105	105
Stationery (markers, batteries, etc.)	68	9
Machetes (depreciation, 2 year life)	253	253
Sundries, including internet	84	134
Total	16,856	19,364
Cost per km^2^ protected	20.1	23.1

^a^The number of targets actually deployed in 2015 and 2016 was 2708, as numbers were slightly increased from the original number of 2600 deployed in 2014 [21]. However, as 3000 targets were ordered, assembled, and paid for each year the full number is included here

### Target checking

For the work in Chad, the targets were deployed and partially checked while conducting the monitoring rounds, especially those after the rainy season. In Uganda, a so-called ‘maintenance’ round which is effectively a second deployment is undertaken [29] but this has not been done in Chad. However, in September 2016 a more formal target checking exercise was undertaken to establish the state of the targets towards the end of the rainy season, seven months after deployment. The costs are given in Table 6. The single mission in 2016 cost USD 5085 or USD 6.1 per km^2^.

**Table 6:** Costs of target checking

Item (percentage of total costs)	2016 (USD)
Specialised equipment (0%)	
None, no targets were replaced	0
Transport (17.4%)	
Vehicle hire	688
Fuel	187
Canoe hire (5 days, several jetties)	8
Staff (66.6%)	
Share of staff salaries	1484
Travel allowances	1906
Local labour (1.0%)	
Supervisor	51
Administrative support (11.8%)	599
Consumables and equipment (3.2%)	
GPS sets (depreciation)	10
GPS sets batteries	17
Fuel for generator	11
Generator maintenance charge	8
Sundries	116
Total	5085
Cost per km^2^ protected	6.1

### Cost summary

Assembling the data from Tables 4, 5, 6,  Tables 7 and 8 summarise the overall costs of the tsetse control work in Chad. The total cost of the activity for 2015 and 2016 averaged at USD 56,133 per year. This comes to USD 66.8 per km^2^ protected and USD 1.4 per person protected for a human population of 39,000, falling somewhat if population increase is assumed.

**Table 7:** Summary of costs for one year’s tsetse control by activity

Activity	Average per year (USD)	% of expenditure	USD/km^2^ protected
Initial tsetse survey^a^	7182	12.8	8.6
Preliminary sensitisation^a^	2220	4.0	2.6
Tsetse monitoring	22,741	40.6	27.1
Target deployment	18,082	32.3	21.6
Target checking	2538	4.5	3.0
Sensitisation	3270	5.8	3.9
Total	56,133	100.0	66.8

^a^20% of the cost of these preparatory activities is attributed to each year’s tsetse control operation

**Table 8:** Summary of costs by category of expenditure

Activity	Average per year (USD)	% of expenditure
Specialised equipment	5129	9.1
Vehicle costs	11,150	19.9
Staff Salaries	13,763	24.5
Staff field allowances	16,856	30.0
Community workers	1408	2.5
Administrative support	5994	10.7
Consumables and equipment	1833	3.3
Total	56,133	100.0

Considering the costs by activity, as would be expected, the bulk of these (85.7%) consist of tsetse surveys, monitoring and target deployment, with sensitisation accounting for nearly a tenth (9.8%) and the remaining 4.5% being for target checking. In terms of cost components, these are dominated by staff costs, with salaries and allowances accounting for 54.5% of costs, the share of transport being 19.9% while specialised equipment, consisting of target, trap and sensitisation materials accounted for only 9.1%.

## Discussion

The cost of USD 66.8 per km^2^ protected is similar to that calculated for the tiny target work in Uganda at USD 85.4. In Chad’s Mandoul focus, with a human population density of 46 people per km^2^ at the start of the project, the cost per person protected at USD 1.4 per year is substantially higher than in Uganda. There a population census was not conducted; however, the high estimated population density of 400–500 per km^2^ implied a cost of only USD 0.2 per person protected [8].

The cost of targets in Mandoul averaged USD 4667 per year, or USD 5.6 per km^2^. This accounted for 25.8% of the cost of the deployment activity but only 8.3% of the cost of all activities. In this cost analysis for Chad, as detailed above, the total cost per target came to USD 1.56, of which 0.33 was the actual recorded costs for freight and insurance and USD 0.23 was for target assembly. In Uganda, the total cost came to USD 1.36, of which USD 0.26 were the recorded costs for target assembly and USD 0.10 was the estimated freight and insurance if targets were sent by sea. The cost of the target fabric and netting was USD 1 in both Chad and Uganda. If the freight and insurance costs for Chad were halved, this would reduce the total cost of targets by 10.6%, but the overall intervention cost by only 0.9%.

Turning to the cost of implementing the target control operation, if all project activities were considered, the total delivery cost per target would be USD 19.0, which compares to USD 11.4 for Uganda. This reflects some of the differences between the two areas, which were primarily due to the remoteness of the Mandoul focus and to the different organisational structure in which the project was embedded. In Arua, some of the deployment and monitoring sites could be accessed by bicycle, others easily by motorcycle or by transporting bicycles on a lorry to the tsetse control areas. In Chad, the swampy terrain meant that bicycles could not be used. Canoes were hired for many operations (Fig. 5). The field work was conducted far from the capital, N’Djaména, where IRED is based. This remoteness may be described in two steps: firstly, the distance of 650 km that needs to be covered from N’Djaména (from where the main part of the team were travelling along with the supplies) to Bodo (the field base where the teams lodged overnight). Secondly, there are the daily movements from Bodo to the river banks, which averaged just over 100 km per day, plus some travel around Bodo itself, typically 20–30 km per day and occasional longer journeys so that the total field trip distance travelled for a deployment was over 2500 km.

In the Ugandan setting, local labour was recruited and the costs for supervision were based on the salaries of a district entomologist. Furthermore, everyone was able to return to the district capital, Arua, every evening. This practice has continued as control in Uganda has scaled up with operations in other districts being managed from the district rather than the national capital. Thus, overnight accommodation and consequently more expensive travel allowances were not needed. In Chad, the per diems paid to staff reflected the fact that staff had to stay overnight in the field and fund their meals while away from N’Djaména. Just over a third of costs were those of running the core organisation, i.e. the administrative support (10.7%) plus a share of the salaries of the staff already employed (24.5%). The remainder, coming out at USD 43.3 per km^2^, is the money that needs to be found in order to implement such a project within the chosen organisational framework.

In both Chad and Uganda, the tsetse population, *G. f. fuscipes* in both areas, was confined to the riverine areas as shown for Chad in Fig. 1. Estimating the treated area is challenging, as it depends on the varying width of the fringing vegetation. The proportion of the area where targets were placed was higher in Chad, due to its greater width. Targets were placed at 20 per linear km in Uganda as against 60 in Chad, thus covering about 144 linear km of river in Uganda in the 500 km^2^ operational area and an estimated 45 km^2^ of riverine vegetation and wetland within the 840 km^2^ focus in Chad [21, 29]. The Mandoul River crossing points (illustrated in Fig. 1) draw in people and livestock, who regularly cross the river for social events, to access local services such as clinics and markets. These are the focal points for disease transmission and explain why HAT cases occur over a large area, defining the size of the focus, and thus the km^2^ protected. Thus, relation to their area of epidemiological impact, the density was 5.7 targets per km^2^ protected in Uganda and 3.2 in Chad.

The riverine environments differed between the two situations. In Uganda there was marked seasonal flooding which washed away a high proportion of targets during the rains, so that after initially replacing these in a target maintenance round as described [8], it was eventually decided simply to undertake two deployments a year. The marshy area of the Mandoul River in Chad, which constitutes the main watercourse in this study, is not subject to seasonal flooding so that there were fewer losses of targets. Hanging the targets rather than planting them in the ground was effective in keeping them in place, and the risk of losses due to the passage of transhumant cattle was effectively mitigated by the sensitisation work. The Mandoul focus is both difficult to access and very challenging to work in during the rainy season. Accordingly, it was felt that a single deployment using a large number of targets was an optimal control strategy. Deployment took place early in the dry season, which lasts from November to May, and tsetse monitoring took place during the dry season in March and May and towards the end of the rains, in October. The targets were thus at their maximum effectiveness during the dry season, when the riverine tsetse flies such as *G. f. fuscipes* are less dispersed, being concentrated in the riverine areas. There was no evidence of a resurgence of flies between deployments in this isolated tsetse population [21]. In this costing, omitting the target checking would reduce costs by 4.5% to USD 63.8 per km^2^, and this practice was subsequently adopted by the project. Simply in terms of costs, it is possible to look at the financial trade-off between a single deployment of large numbers of targets and undertaking two deployments with fewer targets. A full second deployment of 60 targets per km would increase costs by 32.3% to USD 88.4 per km^2^. However, if the number of targets per linear km were reduced to 40 instead of 60, this would also reduce the time spent on deployment, but to the same extent, as the travel from N’Djaména and distances covered along the river would be unaltered. Assuming that this 33% reduction in the number of targets made it possible to reduce all the other deployment costs by 20%, this would reduce the overall cost by 7.5%. However, if the reduced number of targets needed to be combined with a second deployment, the overall cost would increase by 17.3% to USD 78.4 per km^2^. The cost figures as provided in the tables above make it relatively easy to undertake such sensitivity analyses, which can then be assessed relative to their entomological implications.

The sensitisation work in Mandoul accounted for 9.8% of costs and USD 6.5 per km^2^ protected. The targets were well accepted, with no recorded thefts or damage by people and members of the communities clearly understood their purpose, which was reinforced by the CSSE’s. These costs compare with USD 10.0 per km^2^ in Uganda or 11.7% of total costs, including office overheads. In Uganda sensitisation was undertaken as a separate activity which involved visiting villages, training the pre-existing village health teams, meeting villagers and undertaking house-to-house sensitisation with flip charts and targets. In Chad, once the initial visits to villages had taken place, involving village meetings, creating the CSSEʼs and contacting local religious and civic leaders, and materials (posters, leaflets) had been distributed, the follow-up sensitisation focused on radio broadcasts in three languages and relied on the activities of the CSSEs. They were involved in most field missions, and during field work project staff and CSSEs ensured that local people were aware of the activities being undertaken and of their purpose.

Overall, the cost per km^2^ for tiny targets deployed both in Chad and Uganda compares very well with the historical cost of other tsetse control interventions using fixed baits. Based on a synthesis of published field studies [13] these were estimated to cost USD 220–315 per km^2^ protected at 2016 prices in Botswana and Zimbabwe for the classic full size (~1.8 m wide × 1 m high) targets with odour baits deployed against savanna group tsetse flies at a density of 4 per km^2^ protected. For riverine flies, the cost per km^2^ protected at 2016 prices came to about USD 220 per km^2^ for medium sized targets (~ 0.8 m wide × 1.1 m high) and USD 180 for pyramidal traps used against HAT in Côte d’Ivoire and Uganda respectively [13].

This study is one of very few where the full costs were continuously monitored, as compared to each participating institute simply keeping track of its relevant budget lines. In particular the costs included a share of all staff salaries in relation to time spent on the activity. The lessons learnt were, first, that it was important to process and check the cost data after each mission. Secondly, as the data recording on field trips is necessarily conducted by researchers usually more used to collecting entomological data, it is vital to emphasize its purpose and the fact that the number of data items required is relatively small. As the field work progressed, only minor edits were required to the original protocols. The edited versions in both languages are provided in Additional files 1, 2, 3, 4, and the basic data sheet is provided in Additional file 5.

While the cost per km^2^ or person protected indicates its relative cost, in order to fully assess its cost-effectiveness, the costs of controlling tsetse in the Mandoul focus should be ideally related to indicators of its impact. The impact on the tsetse population has been dramatic, with a reduction of 98.6% within two months with only one fly being caught in 2015 and none in 2016 [21]. Evaluating the impact on HAT and AAT has always proved challenging for tsetse and trypanosomiasis control work [30, 31] and is outside the scope of this cost study. For HAT, a number of modelling approaches have been developed recently and applied to estimate of impact of both case-finding /treatment and vector control in number of contexts: Africa-wide in the relation to the need to make an elimination investment case and the 2020 and 2030 goals for HAT elimination [22, 32] and locally for the Boffa focus of Guinea [33] and for the Mandoul focus [21]. The Africa-wide economic analysis [22] incorporated the costs of tiny targets based on the costs calculated for Uganda. For medium and high transmission areas, it concluded that the incremental cost-effectiveness ratios offered by the strategies which included vector control together with new technologies (better diagnostics and improved treatment) were the most attractive. For the Mandoul focus, it was estimated that adding vector control to the case finding and treatment strategies had reduced the number of reported cases in 2014 and 2015 by 39 and 76 cases respectively [21]. This implies immediate savings on treating patients of USD 20,000 and USD 40,000, based on an estimated cost per patient of about USD 500 for the gambiense HAT treatment options in use at that time (derived from published cost values [14, 22] updated to 2016 levels). Such a monetary benefit for those two years would cover a substantial proportion of the cost of tsetse control of USD 56,000 a year. Added to this would be the disability-life years (DALYs) averted due to a reduction in human suffering and mortality from a disease which is fatal in untreated individuals. Ongoing HAT modelling work [32] can also incorporate these new cost data from Chad. Meanwhile the authors conclude that “all models agreed that vector control would consistently avert most infections and likely lead to elimination by 2030 in all considered scenarios” [32]. From the economic point of view, the tsetse control operation in Mandoul has not only prevented HAT cases year by year at a relatively modest cost while implemented, but offers a more rapid end to the need for further expenditure on finding and treating patients, leading to monetary savings as well as DALYs averted occurring beyond the period of the intervention.

Another important consideration in Mandoul is the presence of a substantial sedentary cattle population, estimated at 14,000 head, as described above. The importance of AAT in the Mandoul focus was demonstrated during a survey undertaken in 2012 [23]. The polymerase chain reaction (PCR) analyses showed 18 (13%) of 144 cattle to be infected with *T. vivax*, 13 (9%) with *T. brucei* (*sensu lato*), and 2 (1%) with both. Although *T. brucei* (*s.l.*) are not considered to be pathogenic to cattle, they may include the subspecies *T. b. gambiense* which causes HAT in people in this area. The study undertaken in the Moundou and Sahr areas, some 150 km to the west and east of the Mandoul focus, found serological prevalences of *T. vivax* ranging from 4% to 34% in four cattle herds sampled, with an average prevalence of 14% in 200 cattle [12], thus similar to Mandoul. One animal in this study tested positive for *T. brucei* (*s.l.*) but no cattle in either study were found to have the other main trypanosome pathogenic to cattle, *T. congolense*. For the Mandoul focus, economic benefits in terms of losses averted per bovine per year would need to be of the order of USD 56,000/14,000 to cover fully the vector control costs, equivalent to a productivity increase yielding USD 4 per head per year for the sedentary cattle. There are likely to be additional gains in the transhumant cattle population, which would also have benefitted from the reduced risk of contracting trypanosomiasis while grazing seasonally in the Mandoul area. Collecting data on disease impact in animal populations is complex and time consuming and there have been few recent field studies in Africa and none undertaken in Chad. The existing grey and published literature was reviewed and used to underpin two detailed trypanosomiasis loss mapping studies [34, 35] covering sixteen different cattle production systems for eastern and western Africa. At 2016 prices, the annual production losses from AAT per bovine in the medium oxen use and west African pastoralist systems were modelled as ranging from USD 22–24 [34, 35]. These are the cattle production systems most similar to those in the Mandoul focus, implying that if tsetse control averted only a small proportion of these losses, its costs would be covered and reinforcing the notion of the intervention’s One Health dimension as expressed in previous publications [21, 23].

In order to assess fully the impact of vector control on AAT in Mandoul, a repetition of the cattle trypanosomiasis sampling work undertaken before the intervention started [23] would be an essential step. This would provide valuable extra information on whether trypanosomiasis prevalences have fallen, especially if carried out alongside interviews with sedentary and transhumant cattle keepers to investigate their perceptions of how the disease situation has evolved. In particular, it would be helpful to find out about their current and past use of trypanocides.

## Conclusions

This detailed cost analysis covers the use of tiny targets to control tsetse in the Mandoul sleeping sickness focus of Chad, including pre-intervention activities and two years of continuous cost monitoring. It demonstrates the viability of the cost collection protocol developed and provides a full longitudinal cost dataset covering 13 field missions (initial tsetse survey, sensitisation of local populations, target deployment and checking, tsetse monitoring using traps). The results align with those of an earlier cost study undertaken in Uganda and provide informative contrasts. The Mandoul operation worked out at an annual cost of USD 66.8 per km^2^ protected, similar to that of the tiny target operation in Uganda at USD 85.4 per km^2^. With a much lower population density, based on the census undertaken, the cost per person protected against this potentially fatal disease was USD 1.4 as compared an estimated USD 0.2 in Uganda. The cost per target delivered was higher at USD 19.0 in Chad as against USD 11.4 in Uganda. This largely reflects operational differences: the project being hosted within a local research institute and Mandoul being a remote location, some distance away from project headquarters, with a challenging tsetse habit located in an extensive wetland. Motorised vehicles were often not usable so that canoes were required for transport and even canoes could not access all points. A higher density of traps was used: 60 per linear km as against 20 in Uganda, although the number of linear km treated was lower in Chad, estimated at 45 as against 78 in Uganda. These cost analyses demonstrate that tiny targets are a highly cost-effective method of tsetse control, preventing a deadly neglected tropical disease which also affects livestock, and costing between a quarter and a third of the cost of other target and trap operations used to control tsetse.

## Supplementary information

**Additional file 1: Text S1.** Cost data collection protocol.

**Additional file 2: Text S2.** Cost data collection protocol (in French).

**Additional file 3.** Master cost file - tiny targets.

**Additional file 4.** Master cost file - tiny targets (in French).

**Additional file 5.** Master cost file - Mandoul data (in French).

## Data Availability

The dataset supporting the conclusions of this article is included within the article and in Additional file 5.

## Abbreviations

AAT: animal African trypanosomiasis
CFA: CFA franc of BEAC (Banque des États de lʼAfrique Centrale)
CIRDES: Centre International de Recherche-Développement sur lʼElevage en zone Subhumide
CSSE: Comité de suivi et de sauvegarde des écrans (committee for monitoring and safeguarding of targets)
HAT: human African trypanosomiasis
IRD: Institut de Recherche pour le Développement
IRED: Institut de Recherche en Elevage pour le Développement
LSTM: Liverpool School of Tropical Medicine
PNLTHA: Programme National de Lutte contre la Trypanosomiase Humaine Africaine (Chad)
USD: United States dollar
WHO: World Health Organization
