# Supplementary material for: Delivering ‘tiny targets’ in a remote region of southern Chad: a cost analysis of tsetse control in the Mandoul sleeping sickness focus
Source: Parasit Vectors. 2020 Aug 14;13:419. doi: 10.1186/s13071-020-04286-w (PMC7427721; doi:10.1186/s13071-020-04286-w)
Supplement: Supplementary file 1 — Additional file 1: Text S1. Cost data collection protocol. [file 13071_2020_4286_MOESM1_ESM.pdf]

Additional file 1: Text S1
The use of tiny targets to control tsetse: cost data collection protocol

I Objective

Our aim is to calculate the costs for the 6 component activities of a tiny target control project; these are:

1. Preliminary tsetse monitoring using traps
2. Sensitisation of local populations
3. Target deployment
4. Trap monitoring
5. Target maintenance
6. Office (headquarters) work and administrative support

This will enable us to estimate the cost of such work for:

- Planning future projects (financial resources needed)
- Economic analysis and cost-effectiveness calculations (cost of total resources used up during the work).

It is important to understand that an economic analysis goes beyond simply noting all expenditure.

- We need to include **all the resources required to do the work**. These are usually paid for by several people – the project, the local entomological service, etc.
- Thus the cost for resources used which the' project' does not pay for directly has to be estimated. This would usually be **staff employed by and vehicles owned by and other institutions** but contributing their time to the project. So their time spent on the work, the km travelled by their vehicles and their salaries and allowances will need to be recorded.
- For **durable items**, depreciation is calculated as a measure of how much of that item was 'used up' during the course of the project. So we need to know what the cost of the durable items (vehicles, GPS sets, etc.) was. We then have to estimate their 'useful life' (the number of years they can be used for) to calculate depreciation.
- Some **costs are shared between activities** (such as office work and administration costs). It will be necessary to estimate approximately what these costs are and then to allocate a share to the tiny targets control work.
- In this calculation we are trying to **avoid** including costs that are purely for **research work**. But, if in doubt, it is better to include everything. Then when we do the calculations we can subtract the estimated proportion of expenditure that was just for research work.

II Files for data recording

Expenditure and resource use were recorded in a single Excel file consisting of several worksheets. In addition the vehicles used should each have a **log book** each recording the kilometres travelled and purpose of each journey.

A. Master Cost File

All expenditure relevant to the project should be entered in the master cost file.

For each expenditure item, it should include date, number purchased, unit cost, total cost and, importantly, which activities it was used for or shared between.

For equipment and transport, at the start, it is important to make an inventory of **the initial stock of equipment and resources bought before the start of the work.** This would include vehicles, office and field equipment, consumables (such as stationery, GPS batteries) protective clothing for staff, pangas, etc.

The master cost file contains 6 worksheets.

1. **Activity report.**
   This explains what has been done in each period reported on.
   Productivity indicators should be given to show what has been done (for example traps monitored, targets deployed, villages visited, etc.)

2. **Staff**
   - This is where all staff paid allowances, per diems, travel payments etc. should be noted for all staff as well as the wages received by staff who are paid by the day or for specific work done (such as adding sticks and strings to target cloths).
   - If, in addition, staff have to pay for staying in local accommodation in the field this cost should be entered here.
   - For full-time staff who are employed in-country or abroad, their monthly salaries should be noted separately (as this is private information). Ultimately, in this exercise all staff time inputs were costed at standard local pay rates.
   - To put a value on their time, this worksheet also needs to include a detailed record of field and non-field days worked for every individual working on the project. The easiest way to do this is to work out how many days were needed to prepare each field trip and, after returning from the trip, for process data, samples or for other activities. In addition, the time spent by those managing the project needs to be taken into account.
   - It is possible for costs to be duplicated. An example is if someone is paid a per diem and also notes what they had to spend on accommodation. When this happens, if possible, both costs should be noted. However, the comments need to show that these refer to the same event. When the data is analysed, how the cost should best be presented can be decided.

3. **Vehicles: Fuel and Travel**
   - In this sheet the kilometres travelled for the project by various vehicles should be recorded.
   - In addition the vehicle kilometre reading should be recorded, at least every 6 months, so that it is possible to estimate what percentage of its annual travel is for the project.
   - All fuel costs should entered here. Other vehicle costs go in Sheet 4 below.

4. **Other Vehicle and Transport costs**
   - This sheet should include:

  - the value of all vehicles used for the project
  - the approximate date they were purchased
  - the current price for buying a new vehicle of the same type (the replacement cost).

  Depreciation will be worked out later on the basis of this information.
- It should include all oil, insurance, spare parts, maintenance and servicing costs for vehicles, even if these costs are not directly paid for by the project.
- If vehicles are hired the cost should also be included here.

5. **Equipment and consumables**
   - In this sheet there is no distinction between equipment (from laptops, GPS sets to pangas) and consumables (GPS batteries, notebooks, pencils). The reason is that the dividing line is not always clear nor is it consistently applied by different organisations. Traps and targets should also be included here. For longer lasting equipment items depreciation will be worked out later from the information supplied. In order to facilitate this, the useful life of these items (for example 3 years for a GPS set, 1 year for a target, 10 monitoring field trips for biconical traps). The useful life of an item will vary from project to project according to how much they are used.
   - This sheet should include the cost all the equipment used by the project, but bought for before it starts. The approximate date they were bought should be noted as well as the current price for a new item (the replacement cost). If some things, like overalls or traps, have already been in use, this should be noted in comments.

6. **Office, administration and other office overheads**
   - The nature of these costs varies from country to country. In Uganda, the 'tiny targets' project had its own office however, in Chad it was managed by the Institut de Recherche en Elevage pour le Développement (IRED). Elsewhere, the project administration may be organised differently. So how this work sheet is completed will vary from project to project.

Allocating shares of the work to each activity

- Worksheets 1 to 4 contain columns in which the share of the work which applies to each project activity should be noted. Sometimes this will be very clear – for example target deployment. Sometimes it will not be clear – and an estimate should be made or the column can be left blank. It will be part of the task of the final analysis to distribute the costs between activities. For example vehicle expenditure will be allocated ultimately in relation to the number of KM travelled for each activity.
- **<u>What is vital is that ALL costs are recorded and entered in one of the 6 worksheets.</u>**
- For office overheads, the share is that proportion which is allocated to the WHOLE tiny target project – not its individual sub-activities.

B. Vehicle log books

Each of the vehicles regularly used for the tiny target control work would need to keep a log book when doing work on this project. Government vehicles probably already use one. This would need to record:

- vehicle total kilometres at start of work (so as to know what proportion of the km travelled by that vehicle are for the project)
- kilometres travelled on a particular tiny target activity
- vehicle total kilometres at the end of the activity

It needs to be decided if a log book is needed for each of the trap/target attendants' motorcycles. It will be very important to know how many miles they travel! Also, while mileage recording may not be feasible for bicycles (if they are used), some record of how they have been used and for what activities, needs to be kept.

The information from the vehicle logbooks would then be entered in Sheet (3) of the master cost file.

III Managing the data recording process

It will be important then for one person to be in charge of coordinating any updating of the file. If several people enter data, it is important that this is coordinated and that everyone knows which is the up-to-date version of the file which everyone works on. The updated file will need to be saved with a current date and circulated to those individuals who enter data. A copy should also be sent to the relevant team members and the person who will eventually collate and analyse the data. **The files should always be updated (not started again).**

How often they are updated depends on the work being done. However, when the project is busy it is recommended that they are updated every week. Otherwise it is easy to forget small expenditures and events.

The spreadsheets will definitely need some adjustments and improvements. It is not possible to foresee everything that will be needed. The organisation of the work will be different in each situation.

In particular, it is not exactly clear how ‘office administration and overheads’ will be estimated if there is no dedicated project office. This will need to be determined on a case by case basis.

Whoever is responsible for the process will need to take on the task of chasing people to make sure all expenditure and activities are correctly logged. They will need to decide how often the spreadsheets need to be updated. In any case, costs should be checked and a preliminary analysis undertaken after each field mission.

Experience has shown that the items most likely to cause difficulty in calculating costs or to be omitted are:

- durable items bought for other activities and either brought into the project at its inception from, or shared with other activities (for example tsetse traps, electric generators, camping equipment, etc.;
- the use of different vehicles from different organisations, hence the emphasis on stating which are the vehicles used, who owns them , mileage and who pays the driver for each mission, etc.;
- estimating the total time spent by the different people working on the project, not just during field trips , but before and after field trips and for managing the project.

It is vital to promote a general understanding within the project that the work involved in monitoring costs is not undertaken in order to gratuitously add to peoples’ workload or to check on their performance. There is no advantage in omitting cost data items. Instead, comprehensive and well-presented costs to add value to the project activity and provide a basis for justifying extending this work to other locations and for acquiring funds to do so.
