# Supplementary material for: Delivering ‘tiny targets’ in a remote region of southern Chad: a cost analysis of tsetse control in the Mandoul sleeping sickness focus
Source: Parasit Vectors. 2020 Aug 14;13:419. doi: 10.1186/s13071-020-04286-w (PMC7427721; doi:10.1186/s13071-020-04286-w)
Supplement: Supplementary file 2 — Additional file 2: Text S2. Cost data collection protocol (in French). [file 13071_2020_4286_MOESM2_ESM.pdf]

Additional file 2: Text S2

Utilisation des écrans de petite taille dans la lutte contre les glossines : Protocole de collecte des données sur les coûts

I. Objectif

L'objectif est de calculer les coûts de 6 principales activités menées dans le cadre d'une lutte contre les glossines par l'utilisation d'écrans de petite taille, que sont :

1. L'enquête entomologique de base par l'utilisation de pièges
2. La sensibilisation des communautés locales
3. Le déploiement des écrans
4. Le suivi/évaluation des densités de glossines
5. L'entretien des écrans
6. Le travail de bureau (siège) et le soutien administratif

Cela permettra l'estimation des coûts de ces activités pour :

- La planification future des projets (les ressources financières nécessaires)
- L'analyse économique et le calcul coût-efficacité (coût total des ressources utilisées pour la mise en oeuvre du projet)

Il est important de comprendre qu'une analyse économique va au-delà d'une simple notification de toutes les dépenses d'un projet.

- Il y a un besoin d'inclure **toutes les ressources nécessaires pour faire le travail.** Celles-ci sont généralement financées par plusieurs personnes, le projet, le service local d'entomologie, etc.
- Ainsi, les coûts des ressources utilisées, mais qui ne sont pas financées directement par le projet doivent être estimés. En général, il s'agit soit du **personnel d'autres institutions** mais dont une partie du temps est consacré au projet **et/ou des véhicules appartenant à d'autres institutions** mais en partie utilisés dans le cadre du projet. Ainsi, le temps utilisé pour le travail, le kilométrage des véhicules, les salaires et perdiems devront être notés.
- Pour les **objets durables**, l'amortissement est calculé pour estimer le coût de l'utilisation de cet objet au cours du projet. Ainsi, il est nécessaire de connaître quels étaient les coûts de ces objets durables (véhicules, GPS, kits, etc.). Par la suite, leur durée de vie sera estimée (nombre d'années pendant lesquelles ils peuvent être utilisés) pour calculer l'amortissement.
- Certains coûts sont **partagés entre les activités** (tels que le travail au bureau et le soutien administratif). Il est nécessaire d'estimer approximativement leurs coûts et allouer une partie au projet de lutte.
- Dans ce travail, nous essayons d'**éviter** d'inclure les coûts qui sont purement liés **aux travaux de recherche**. En cas de doute cependant, il y a lieu de les inclure tous. Les dépenses faites seulement pour les travaux de recherches seront par la suite soustraites au moment des calculs.

II. Les fichiers pour l'enregistrement des données

Les dépenses et l'utilisation des ressources ont été enregistrées dans un même fichier Excel, comportant plusieurs feuilles de calcul. En plus, les véhicules devraient avoir un **carnet de bord** où seront marqués le kilométrage et l'objectif de chaque déplacement.

A. Le fichier principal des coûts

Toutes les dépenses liées au projet devront être entrées dans ce fichier principal.

Pour chaque acquisition, on note la date, le nombre d'objets achetés, le coût unitaire, le coût total et surtout les activités pour lesquelles cet objet a été utilisé.

Pour les équipements et le transport, il est important au départ, de faire l'inventaire **du stock initial des équipements et ressources acquis avant le projet.** Cela inclura les véhicules, l'équipement de bureau et de terrain, les consommables (bureautique, piles de GPS par exemple), les habits de protection des agents, les machettes, etc.

Le fichier principal des coûts contient 6 feuilles de calcul.

1. Le rapport d'activités

Il explique ce qui a été fait au cours de chaque période rapportée. Des indicateurs de productivité devraient être donnés pour montrer ce qui a été fait (par exemple les pièges suivis, les écrans déployés, les villages visités, etc.).

2. Le personnel

- C'est là où les indemnités, les perdiems, les frais de voyages, etc. doivent être notés pour tout le personnel de même que les prestations payées aux journaliers pour un travail spécifique fait (les assembleurs d'écrans par exemple)
- Si, en plus, le personnel doit payer pour passer la nuit sur le terrain ce coût devrait être noté ici.
- Pour le personnel permanent qui est employé au pays ou à l'extérieur, le salaire mensuel devrait être noté séparément (comme c'est une information privée). A la fin, dans ce calcul fait ici, le coût du temps consacré au projet par tout le personnel a été estimé sur la base des salaires au niveau local.
- Pour mettre une valeur à leur temps, cette feuille inclura également une notification détaillée des jours de terrain et des jours de non-terrain pour toute personne travaillant dans le projet. Pour ce faire, le plus simple est de noter combien de jours ont été nécessaires pour préparer chaque mission de terrain et, au retour de la mission de terrain, de noter le nombre de jours nécessaires pour analyser les données, les échantillons ou pour toutes autres activités. Ajouter à cela, il faudra tenir compte des personnes qui ont passé du temps sur la gestion du projet.
- Il est possible que des coûts soient doublement comptabilisés. Un exemple serait qu'une personne perçoive des perdiems et marque également ce qu'elle a dépensé pour son logement. Si le cas arrivait et si c'est possible, les deux coûts devraient être notés. Mais, les commentaires devraient montrer qu'il s'agit bien du même événement. Pendant l'analyse des données, on pourra décider où la dépense serait la mieux présentée.

3. Les véhicules : carburant et déplacements

- On notera sur cette feuille les kilomètres parcourus pour le projet par les différents véhicules.
- Le kilométrage devrait être noté régulièrement, ainsi, il sera possible d'estimer quel pourcentage des déplacements est lié au projet.
- Tous les frais de carburant devraient être notés sur cette fiche. Les autres frais des véhicules seront mentionnés sur la feuille 4.

4. Autres frais de véhicules et de transport

- Cette feuille devra inclure :
  - la valeur estimée de tous les véhicules utilisés pour le projet
  - leur date approximative d'acquisition
  - le prix actuel d'un nouveau véhicule du même type (le coût de remplacement).

Leur amortissement sera estimé sur la base de ces renseignements.

- Elle inclut aussi l'huile, l'assurance, les pièces de rechange, la maintenance et les frais de service des véhicules, même si ces frais ne sont pas payés directement par le projet.
- Le coût des véhicules loués doit aussi être inclus ici.

5. **Equipements et consommables**
    - Sur cette feuille, on ne distingue pas équipement (ordinateurs, GPS, machettes, etc.) de consommables (piles de GPS, bloc-notes, stylos, etc.). La raison est que la ligne qui les sépare n'est pas toujours claire et beaucoup d'institutions ne l'appliquent pas. Les pièges et les écrans devraient être inclus ici. L'amortissement des équipements à longue durée de vie sera calculé plus tard à partir de ces informations. Pour faciliter cela, il faudrait également indiquer la vie utile approximative de ces équipements (par exemple 3 ans pour un ensemble GPS, 1 an pour un écran, 10 missions de suivi pour les pièges biconiques). Ces durées de vie varieront selon le projet et l'intensité de leur utilisation.
    - Les coûts de tous les équipements utilisés par le projet, mais achetés avant son démarrage devraient être notés ici. Leur date approximative d'achat devrait être mentionnée ainsi que leur prix d'achat actuel (le coût de remplacement). Si des équipements, (comme par exemple les tenues de protection et les pièges) sont déjà utilisés, cela doit être indiqué dans les commentaires.

6. **Dépenses administratives et de bureau**
    - La nature de ces coûts varie d'un pays à un autre. En Ouganda, le projet « petits écrans » avait son propre bureau mais au Tchad, la gestion du projet se faisait par l'Institut de Recherche en Elevage pour le Développement (IRED). Ainsi, le contenu de cette feuille variera d'un projet à un autre.

Partage des coûts par activité

- Les feuilles 1 à 4 contiennent des colonnes dans lesquelles la part de travail qui s'applique à chaque activité du projet doit être notée. Parfois, cela sera très clair, comme par exemple le déploiement des écrans. Parfois, ce ne sera pas clair, et une estimation devrait être faite ou la colonne laissée vide. Le partage des coûts entre activités se fait au cours de l'analyse finale. Par exemple, les dépenses des véhicules seront allouées en fonction du nombre de kilomètres parcourus pour chaque activité.
- **Ce qui est important est que TOUS les coûts soient notés dans une des ces 6 feuilles.**
- Pour les frais de bureau, la part est la proportion allouée au projet ENTIER et non à la sous – activité individuelle.

B. Carnets de bord des véhicules

Chaque véhicule utilisé régulièrement dans le cadre du projet devrait avoir un carnet de bord. Les véhicules du gouvernement en ont déjà certainement. On y notera :

- le kilométrage total au début du travail (ainsi, on saura quelle proportion de kilomètres parcourus par ce véhicule est liée au projet).
- les kilomètres parcourus pour chaque activité particulière du projet
- le kilométrage total du véhicule à la fin de l'activité

On a besoin de décider s'il faut un carnet de bord pour chacune des motos des surveillants des pièges et écrans. Il est très important de savoir combien de kilomètres ils parcourent. Egalement, s'il est bien difficile de noter la distance parcourue par les bicyclettes, une notification sur comment elles ont été utilisées pour telle ou telle activité est par contre nécessaire.

Les informations contenues dans les carnets de bord des véhicules seront par la suite notées dans la feuille 3 du fichier principal des coûts.

III. Gestion de la procédure d'entrée des données

Il est important qu'une seule personne soit responsable pour la coordination de la mise à jour du fichier. Si plusieurs personnes font entrer les données, il est important que cela se fasse de manière coordonnée et que chacun sache quelle est la dernière version du fichier sur laquelle on travaille. Le fichier à jour devrait être enregistré avec la date du jour et mis en circulation entre ceux qui entrent les données. Une copie doit également être envoyée aux membres de l'équipe concernés et à la personne qui finira par rassembler et analyser les données. **Les fichiers doivent être toujours mis à jour et non repris de nouveau.**

La fréquence de la mise à jour dépend du travail en cours. Cependant, si le projet est en pleine activité, il est recommandé que le fichier soit mis à jour chaque semaine. Autrement, il est facile d'oublier des petites dépenses et évènements.

Les fiches auront certainement besoin d'être adaptées et améliorées, étant donné qu'il est difficile de voir par avance tout ce qui est utile à noter. L'organisation du travail sera différente dans chaque situation.

En particulier, l'estimation des coûts liés à l'administration du bureau n'est pas claire s'il n'y a pas de bureau dédié au Projet. Cela aura besoin d'être déterminé cas par cas.

Toute personne responsable du processus aura besoin de s'assurer que toutes les dépenses et activités sont correctement notées. Elle décidera de la fréquence de mise à jour des fiches. Dans tous les cas, les coûts doivent être vérifiés et une analyse préliminaire entreprise après chaque mission sur le terrain.

L'expérience a montré que les éléments les plus susceptibles d'entraîner des difficultés de calcul des coûts ou d'être omis sont les suivants:

- articles durables déjà achetés pour d'autres activités et introduits dans le projet ou articles durables partagés avec d'autres activités (pièges à tsé-tsé, générateurs électriques, matériel de camping, etc.);
- l'utilisation de différents véhicules appartenant à différentes organisations, d'où l'importance donnée à la description des véhicules utilisés, de noter leur propriétaire, au kilométrage parcouru et au paiement du conducteur pour chaque mission, etc.;
- calcul du temps total investi par les différentes personnes travaillant sur le projet, non seulement au cours des missions au terrain, mais avant et après ces missions et pour gérer le projet.

Il est essentiel d'encourager une compréhension générale au sein du projet selon laquelle ce travail de suivi des coûts ne vise pas à alourdir gratuitement la charge de travail des personnes ni à contrôler leurs performances. L'omission d'éléments des coûts ne présente aucun avantage. Par contre, des coûts complets et bien présentés ajoutent de la valeur à l'activité du projet et produisent des informations qui justifieront l'extension de cette activité à d'autres endroits et le financement de telles activités.
